# Influence of Sub-Inhibitory Concentrations of Sanitizers and Oxacillin on the Resistance of Methicillin-Resistant *Staphylococcus* spp.

**DOI:** 10.3390/vetsci12100979

**Published:** 2025-10-11

**Authors:** Maria Eugênia Betim, Daniel Lucino Silva dos Santos, Thiago dos Santos Lopes, Bruna Lourenço Crippa, Érika Romão Bonsaglia, Stéfani Thais Alves Dantas, Vera Lúcia Mores Rall, Fernanda Buzzola, Julia Arantes Galvão, Clarice Gebara, André Thaler, Nathália Cristina Cirone Silva

**Affiliations:** 1 Department of Food Sciences and Nutrition, Faculty of Food Engineering, Universidade Estadual de Campinas (UNICAMP), Campinas 13083-862, Brazild255068@dac.unicamp.br (D.L.S.d.S.); t244718@dac.unicamp.br (T.d.S.L.); lourencobruna@yahoo.com.br (B.L.C.); 2 Department of Animal Nutrition and Production, School of Veterinary Medicine and Animal Sciences, University of São Paulo (USP), Pirassununga 13635-900, Brazil; erikabonsaglia@usp.br (É.R.B.); stefani.dantas@usp.br (S.T.A.D.); 3 Department of Genetics, Microbiology and Immunology, Institute of Biosciences, São Paulo State University, Botucatu 18618-691, Brazil; vera.rall@unesp.br; 4 Faculty of Medicine, Institute of Research in Microbiology and Medical Parasitology (IMPAM), National Scientific and Technical Research Council (CONICET), University of Buenos Aires (UBA), Buenos Aires C1053ABH, Argentina; ferbuz@fmed.uba.ar; 5 Department of Veterinary Medicine, Federal University of Paraná (UFPR), Curitiba 80035-090, Brazil; julia.galvao@ufpr.br; 6 Department of Veterinary Medicine, Federal University of Goiás, Goiânia 74605-080, Brazil; claricegebara@ufg.br; 7 Center for Agro-Veterinary Sciences, Santa Catarina State University, Lages 88520-000, Brazil; andre.thaler@udesc.br

**Keywords:** sanitizers, MRNAS, bacterial resistance

## Abstract

Methicillin-resistant *Staphylococcus* spp. are microorganisms found in dairy products, bovine mastitis, and human infections. The prevalence of resistant strains from this genus in the food chain is increasing, drawing attention to transmission in the community and highlighting the importance of One Health studies. Thus, the aim of this study was to determine the MIC of oxacillin (OXA) and the sanitizers benzalkonium chloride (BAC) and sodium hypochlorite (HP) against isolates of methicillin-resistant *Staphylococcus* spp., and to evaluate the possible influence of sub-MIC application of these compounds on bacterial cells, in order to observe possible microbial resistance. Ten isolates of methicillin-resistant *Staphylococcus* spp. (*S. epidermidis* and *S. chromogenes*) were used. Among the sanitizers, BAC showed greater efficiency during the pre-inhibition test. Increased resistance to OXA was found in isolates of *S. chromogenes* and *S. epidermidis* after sub-MICs of 50% and 90% of OXA, while sub-inhibition of HP favored resistance to OXA. The application of HP and OXA, even at low concentrations, induced a reduction in biofilm production. This study shows that sub-inhibitory sanitizer exposure in *Staphylococcus* spp. induces antimicrobial resistance phenotypes linked to mutations in regulatory, mobile, and DNA repair genes. These findings suggest that selective pressure promotes resistant variants through genomic plasticity and regulatory activation, supporting the hypothesis that sanitizer residues may drive multidrug resistance emergence, although further functional validation is required.

## Simple Summary

This study evaluated the impact of applying sub-inhibitory concentrations (sub-MICs) of benzalkonium chloride (BAC), sodium hypochlorite (HP), and oxacillin (OXA) on *Staphylococcus* spp. isolates. The different minimum inhibitory concentrations (MICs), antimicrobial resistance, biofilm formation, and changes in gene expression were observed through whole genome sequencing. Among the disinfectants, BAC was the most effective in tests prior to sub-MIC applications. OXA resistance increased in *S. chromogenes* and *S. epidermidis* isolates after 50% and 90% OXA sub-inhibitions, and sub-MIC HP may favor OXA resistance. HP also induced a reduction in biofilm production. Overall, exposure of the strains to the disinfectants caused changes in the gene expression of the evaluated bacteria.

## 1. Introduction

*Staphylococcus* spp. is one of the main bacterial genera involved in subclinical bovine mastitis, whose main pathogen is the *S. aureus* species, recognized worldwide as a cause of foodborne illness and human and animal infections such as bacteremia, wound infections, and mastitis [1,2]. This microorganism is also considered one of the most important human pathogens due to its ability to express a wide range of virulence and resistance mechanisms [3,4]. In addition to the aforementioned species, non-aureus strains of the genus *Staphylococcus* (NAS), which include mostly coagulase-negative staphylococci (CNS), are frequently studied and commonly isolated from dairy products [1]. Although less virulent than *S. aureus*, NAS are responsible for both cases of mastitis and infections in humans [5,6]. Their presence in dairy herds can compromise milk quality, leading to significant economic losses for the dairy industry, as well as representing a food safety concern, as milk and its derivatives can serve as vehicles for transmitting these microorganisms [7].

Recent studies highlight the growing importance of NAS in the context of One Health, given their wide distribution in humans, animals, and the environment, and their role as reservoirs of antimicrobial resistance and virulence genes. From this group, *S. chromogenes* and *S. epidermidis* stand out. *S. chromogenes* is one of the most prevalent species in bovine subclinical mastitis, particularly in heifers, and has been associated with persistent intramammary infections, increased somatic cell counts, and substantial economic impacts on milk production systems [7,8,9]. Additionally, strains involved in persistent infections can induce a marked inflammatory response, characterized by the production of cytokines such as IL-17A and IFN-γ, demonstrating their capacity to modulate the bovine immune system [10]. On the other hand, *S. epidermidis*, although traditionally regarded as a skin commensal, has emerged as a relevant opportunistic pathogen. It can cause subclinical intramammary infections in cattle, form biofilms on medical devices, and act as a reservoir of antimicrobial resistance genes, with potential transmission across species [7,11].

Currently, methicillin resistant *Staphylococcus aureus* (MRSA) and methicillin-resistant non-*aureus Staphylococcus* (MRNAS) are distributed worldwide, and their ability to acquire antibiotic resistance mechanisms raises concerns for public health [4]. Studies have shown the distribution and isolation of MRSA strains at various stages of raw material procurement, food processing and distribution [6,12]. This indicates that the food production chain can be a route of transmission and a vehicle for these resistant microorganisms to reach humans [3,13].

In addition to concerns about antibiotic resistance, *Staphylococcus* is capable of developing resistance to other antimicrobials, such as sanitizers. This resistance to sanitizers has emerged rapidly and is frequently reported, largely due to the misuse and overuse of disinfectants, which apply selective pressure, and lead to the emergence of resistant cells through vertical and horizontal gene transfer [14]. Furthermore, bacteria can acquire resistance to biocides due to long-term sublethal dose exposure and, subsequently, transfer the acquired resistance genes, thus facilitating the spread of resistance genes between bacteria in vitro [15].

Therefore, this study aims to determine the inhibitory concentrations of oxacillin (OXA) and sanitizers routinely used in industry sanitization processes (benzalkonium chloride (BAC) and sodium hypochlorite (HP)) and to evaluate the potential influence of sub-inhibitory concentrations on the induction of antimicrobial resistance in bacterial cells of methicillin-resistant *Staphylococcus* spp. The effects of sub-MIC on biofilm production and gene expression in bacteria after treatment were also evaluated.

## 2. Materials and Methods

### 2.1. Microorganisms and Antimicrobial Agents

The isolates included six *S. epidermidis* (19A, 32A, 32B, 126B, 178B, 181B) and four *S. chromogenes* (212A, 41B, 66B, 250B). The isolates were obtained from a previous study [11] that analyzed milk samples from mammary quarters of subclinical and clinical mastitis cases. The bacterial species were identified using Matrix-Assisted Laser Desorption/Ionization Time-of-Flight Mass Spectrometry (MALDI-ToF MS), and the presence of the *mecA* gene was previously detected by Polymerase Chain Reaction (PCR) [16,17].

The HP (molar mass (MM) = 74.50 g/mol, 5% active chlorine) and the aqueous BAC solution (MM = 0.004/mol) were purchased from Dinâmica Química Contemporânea Ltda (Dinâmica^®^, Indaiatuba, SP, Brazil). OXA (purity ≥ 95%, MM = 423.42 g/mol) was purchased from Sigma (Sigma-Aldrich^®^, São Paulo, SP, Brazil).

### 2.2. Determination of Minimum Inhibitory Concentration (MIC)

The MIC of the evaluated sanitizers was determined by microdilution in trypticase soy broth (TSB—KASVI^®^, Pinhais, PR, Brazil) using 96-well plates for BAC and HP, following the method described by Abreu et al. [4]. The MIC for OXA was determined according to the Clinical and Laboratory Standards Institute CLSI [18] protocol, with adaptations. Initially, all isolates were inoculated into brain heart infusion broth (BHI—KASVI^®^, Pinhais, PR, Brazil) and incubated at 37 °C for 24 h. The cultures were then standardized to the McFarland 0.5 scale (1.5 × 10^8^ CFU/mL). Subsequently, 180 µL of serial dilutions of either disinfectants or OXA in TSB were dispensed into the wells, starting at an initial concentration of 1000 ppm. Twelve concentrations were tested using 1:2 serial dilutions. A 1000 ppm stock solution was prepared in sterile distilled water and diluted down to 0.49 ppm. Afterward, 20 µL of the standardized bacterial suspension (1 × 10^8^ CFU/mL) was added to each well, resulting in a final inoculum of 1 × 10^7^ CFU/mL per well and a final volume of 200 µL.

Plates were incubated at 37 °C for 5, 10, and 15 min, as well as for 24 h. Following incubation, disinfectants were neutralized by transferring 10 µL from each well to a second 96-well plate containing 90 µL of neutralizing broth. For BAC, Letheen broth (Merck, Darmstadt, Germany) supplemented with 0.5% Tween 80 was used, and for HP, TSB supplemented with 1% sodium thiosulfate was used. These plates were then incubated at 37 °C for 24 h [4]. Negative controls were prepared in the same way but without bacterial inoculation, while positive controls were prepared by replacing disinfectants and OXA with ultrapure water.

In wells treated with disinfectants, 50 µL of 0.01% resazurin solution was added and incubated at room temperature for 5 to 10 min. Results were recorded visually, as follows: wells that remained blue indicated bacterial inhibition and defined the MIC, while wells that turned pink indicated bacterial growth [19]. For OXA, MIC determination was also based on visual observation, with the absence of turbidity indicating growth inhibition.

### 2.3. Effect of Sub-MIC Antimicrobials on Growth Characteristics, Susceptibility to Sanitizers and OXA

The sub-MICs of the sanitizers and OXA were established based on the MIC values determined in Section 2.2. The experiments were conducted as previously described [20], with modifications, using 24 h as the contact time. The initial inoculum for the isolates was standardized at 1.5 × 10^8^ CFU/mL, and the sub-MIC of each antimicrobial was added at concentrations of ¼ of the MIC 50 and ¼ of the MIC 90 of each initial MIC. The treatment number and concentrations (in ppm) of each antimicrobial agent in the sub-MIC dose applications are shown in Table 1.

In all tubes showing bacterial growth after the respective contact time, an aliquot of 50 μL was transferred to a freshly prepared tube containing the same concentration of each sanitizer and incubated again. If growth was observed, the cultures were re-exposed to the same sub-MIC conditions in subsequent cycles, until they had been subjected to sub-inhibitory concentrations for a total period of up to 72 h. After this process, a portion of the tube was spread on a brain heart infusion plate (BHI, KASVI^®^) and incubated at 37 °C for 24 h to isolate the surviving microorganism.

### 2.4. Disk Diffusion Assay

Antibiograms were performed on Müller–Hinton agar (KASVI^®^) plates. With the aid of a swab, the strains were spread onto the plate surface at a concentration equivalent to a 0.5 McFarland standard (1 × 10^8^ CFU/mL). The antibiotics tested were as follows: gentamicin (10 µg) (GEN), oxacillin (1 µg) (OXA), cefoxitin (30 µg) (CEF), tetracycline (30 µg) (TET), erythromycin (15 µg) (ERI), tobramycin (10 µg) (TOB), penicillin (10 µg) (PEN), streptomycin (10 µg) (EST), clindamycin (2 µg) (CLIN), and chloramphenicol (30 µg) (CLO). The plates were incubated at 35 °C for 24 h, after which the resulting inhibition halos were measured with a caliper. Reference values for the halo diameters, which indicate whether a strain is resistant or sensitive, were obtained following the methods of the Clinical and Laboratory Standards Institute (CLSI) [21] and The European Committee on Antimicrobial Susceptibility Testing (EUCAST) [22]. Subsequently, the strains that showed growth after sub-inhibition treatment (as described in Section 2.3) were sub-cultured (initially inoculated in BHI broth and incubated at 37 °C for 24 h, and then diluted to a McFarland standard of 0.5) and re-tested with a new antibiogram to compare the results with previous ones.

### 2.5. Biofilm Quantification Assay

For quantification of biofilm formation, each isolate, before and after sub-inhibition treatment, was inoculated into BHI broth and incubated at 37 °C for 24 h. The cultures were then diluted with BHI broth to a 0.5 McFarland standard (approximately 1.5 × 10^8^ CFU/mL). Then, a 200 µL aliquot was plated in triplicate in 96-well microplates. The isolates were incubated for 48 h without shaking, and the BHI broth was changed every 24 h. After the incubation period, the plates were washed three times with buffered saline solution (PBS, pH 7.4), dried at room temperature, and stained with 1% crystal violet for 15 min. After three washes with distilled water, the biofilm was resuspended in 200 µL of glacial acetic acid (33% *v*/*v*) for 10 min, and the plates were read in a microplate spectrophotometer at a wavelength of 570 nm. Uninoculated BHI was used as a blank, and the absorbance value was corrected by subtracting the average of the blank wells. *S. aureus* subsp. *aureus* Rosenbach ATCC 6538 and *S. epidermidis* ATCC 35928 were used as a positive control, while *S. epidermidis* ATCC 12228 served as the negative control.

The strains were categorized as follows—non-adherent, weakly adherent, moderately adherent, or strongly adherent—based on the optical density (OD) values of the bacterial biofilms. The cutoff OD (ODc) was defined following the method of Stepanovic et al. [23], according to the following equations:

DO≤DOc :Non−adherent (NA)

DOc<DO≤2 ∗ DOc :Weakly adherent (WA)

2 ∗ DOc<DO≤4 ∗ DOc :Moderately adherent

4 ∗ DOc<DO :Strongly adherent

### 2.6. Whole Genome Sequencing (WGS)

Genomic DNA was extracted for sequencing using the commercial DNEasy^®^ PowerFood Microbial Kit (QIAGEN, Hilden, Germany) following the manufacturer’s protocol. Genome sequencing of each isolate was conducted at the Brazilian Center for Research in Energy and Materials (CNPEM) using Illumina technology. A library was generated on the NextSeq platform (https://www.illumina.com/systems/sequencing-platforms/nextseq.html, accessed on 14 April 2025) according to its protocol. The quality of the obtained sequences was verified using the FastQC tool (https://github.com/s-andrews/FastQC, accessed on 14 April 2025), followed by genome assembly using the Unicycler tool (https://github.com/rrwick/Unicycler, accessed on 14 April 2025) [24]. The quality of the assembly was analyzed following recommendations from Gurevich et al. [25], and possible contaminations were checked using Kraken2 (https://github.com/DerrickWood/kraken2, accessed on 14 April 2025) [26]. Isolates showing significant changes in MIC before and after sub-inhibition were selected for this stage: 19A, 19A2, 32a, 32a4, 181B, 181B2, 212a, and 212a4.

The analysis of the resulting pangenome allowed for the detection of Single Nucleotide Polymorphisms (SNPs) obtained in Snippy (v4.6.0) (https://github.com/tseemann/snippy, accessed on 14 April 2025). To identify the genes where these mutations occurred, the coordinates of the mutations were mapped to a reference genome. By consulting three genomes per isolate, it was possible to determine the specific gene in which each mutation was located. Next, the functional annotation information was extracted, including the name and function of the gene, as well as the encoded protein, as described in the genomic records of the National Center for Biotechnology Information (NCBI).

### 2.7. Statistical Analysis

The mean values, standard deviations and Tukey’s analysis of variance (ANOVA) (*p* ≤ 0.05) were obtained using Sisvar 5.6 software (Universidade de Lavras, Lavras, Brazil). The figures were generated using GraphPad prism software version 8.0.1 (Dotmatics, Boston, MA, USA).

## 3. Results

### 3.1. Determination of the MIC of Antimicrobial Agents Against Staphylococcus spp. Before and After Application of Sub-MIC Doses

**Figure 1:**
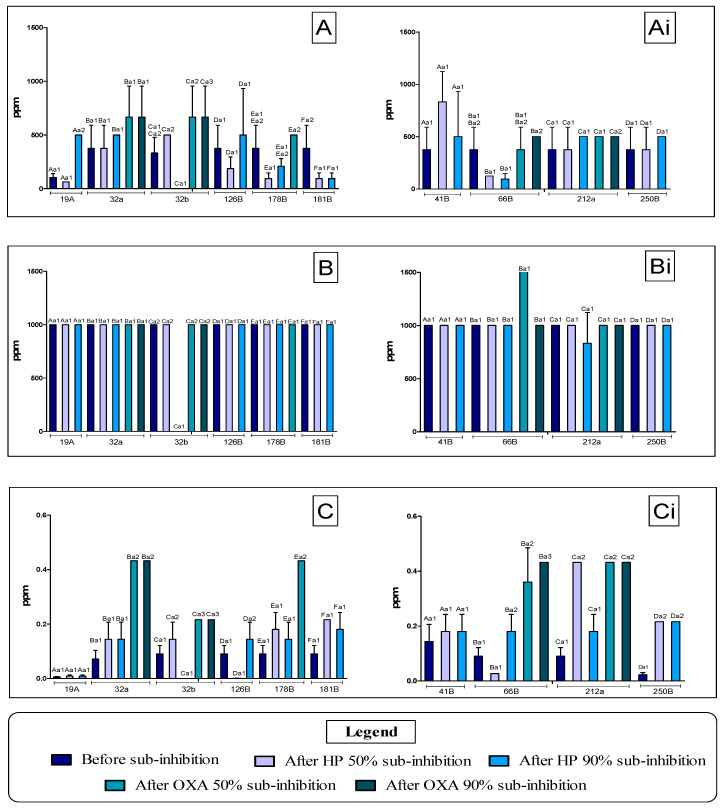
Graphs of the minimum inhibitory concentration (MIC) values before and after the application of sodium hypochlorite (HP), benzalkonium chloride (BAC) and oxacillin (OXA) at sub-inhibitory concentrations against non-*aureus Staphylococcus*. (**A**): Effect of BAC against *S. epidermidis* before and after sub-inhibition. (**Ai**): Effect of BAC against *S. chromogenes* before and after sub-inhibition. (**B**): Effect of HP against *S. epidermidis* before and after sub-inhibition. (**Bi**): Action of HP against *S. chromogenes* before and after sub-inhibition. (**C**): Effect of OXA against *S. epidermidis* before and after sub-inhibition. (**Ci**): Effect of OXA against *S. chromogenes* before and after sub-inhibition. Equal lowercase letters on the columns indicate significant equality by Tukey (*p* < 0.05).

After MIC analysis of antimicrobial agents against NAS isolates, the results of the sanitizers and OXA are shown in Figure 1.

For the *S. epidermidis* isolates, as shown in Figure 1 A, the MIC values ranged from 104.16 to 375.00 ppm for BAC, with greater sensitivity to the sanitizer noted for isolate 19A. For the application of 50% and 90% concentrations of HP and OXA, isolates 32A and 32b showed MICs equal to 666.66 ppm, higher values after sub-inhibition. When HP was applied, the MIC values were 1000 ppm, with no variation in the applications after the sub-inhibition test. For OXA, isolate 19A was the most sensitive with an MIC of 0.005 ppm, while isolates 32A and 178B had MICs of 0.432 ppm for the antimicrobial.

Analyzing the MIC values against *S. chromogenes* in BAC applications, the isolates gave a value equal to 375 ppm, while for the sub-inhibition test, isolate 41b showed the highest MIC value, 833.33 ppm, using a concentration of 50% hypochlorite. Using HP, the isolates showed MICs of 1000 ppm before and after sub-inhibition, except for isolate 66B, which showed MICs of 1666 ppm after sub-inhibition with OXA at a concentration of 50%. The MIC values for OXA ranged from 0.022 to 0.432 ppm. It should be noted that after sub-inhibition the highest values of 0.360 to 0.432 are observed, respectively, in isolates 66B and 212a after testing with sub-inhibition at a concentration of 50% and 90% for OXA.

As shown in Box S1 (Supplementary Materials), with contact times of 5, 10, and 15 min for the sanitizers and OXA against *Staphylococcus* strains, the antimicrobial activity of HP improved significantly between 5 and 10 min, where the mean MIC values at 5 and 10 min were 138 and 99 ppm, respectively. For BAC, specific isolates (e.g., 66B, 41B, and 250B) showed a significant decrease in mean MIC values (from 109 ppm to 67 ppm), indicating greater inhibition after 15 min. OXA was highly effective for most isolates, especially those with MIC equal to 0.000105. However, isolates 32A and 32B had higher MIC values (0.001740 ppm).

### 3.2. Agar Diffusion Disk Analysis

Tables S1–S3 (Supplementary Materials) show the antibiotic resistance profile of the *Staphylococcus* strains before and after sub-inhibitory exposure to sanitizers and OXA. The results demonstrate that some strains developed higher levels of resistance. In general, the strains with the highest number of resistance were those in group 32A (32A3, 32A4, 32B3, and 32B4, all *S. epidermidis*), which showed resistance to nine of the ten antibiotics tested, characterizing a multidrug resistance profile. Before sub-inhibition, the strain was already resistant to five antibiotics; after the test, it acquired resistance to CEF, TOB (for 32A3 and 32A4), and EST. Other strains, such as 32B1, 178B1, 181B1, and 212A2, showed resistance to six antibiotics, while strain 212a (*S. chromogenes*) was sensitive to all antibiotics before sub-inhibition. After the test, acquired resistance to five antibiotics (CEF, TET, ERI, CLI (in 212A3 and 212A4), and PEN) was observed. In contrast, strains like 41B, 66B, 178B, 181B, and 250B were mostly sensitive before sub-inhibition and maintained their sensitivity profile after the tests.

When evaluating the antimicrobial susceptibility profiles of bacterial strains using the interpretive criteria established by CLSI and EUCAST, a marked variation was observed between the two systems, with EUCAST generally assigning higher resistance rates. According to CLSI, resistance rates ranged from 0%, as observed for GEN, OXA, TOB, and CLO, to 55.2% for CEF. In contrast, EUCAST yielded higher resistance rates for most antimicrobials, ranging from 6.9% for TOB to 75.9% for EST. For example, OXA showed 0% resistance under CLSI, whereas EUCAST classified 55.2% of the isolates as resistant. Similarly, GEN was considered susceptible in 100% of isolates according to CLSI, but 24.1% were classified as resistant by EUCAST. PEN resistance was observed in 41.4% of strains under CLSI versus 69.0% under EUCAST. ERI showed resistance in 44.8% of isolates with CLSI and 65.5% with EUCAST, while CLI resistance was reported in 24.1% and 41.4% of isolates, respectively. The greatest discrepancy was noted for EST, with 41.4% resistance reported using CLSI criteria compared to 75.9% using EUCAST.

### 3.3. Quantification of Biofilm Formation

The evaluation results for biofilm formation and adhesion by *Staphylococcus* strains are shown in Table 2. It should be noted that six strains that were originally classified as weakly adherent (WA) became non-adherent (NA) variants after treatment (19A, 32B, 41B, 66B, 212A, and 250B). In some cases, the loss was partial, meaning only some variants became non-adherent. Conversely, three strains that were initially non-adherent (NA) began to generate adherent variants (WA) after treatment (32A, 126B, and 178B).

For the effect of OXA at ¼ MIC50 applications, after sub-inhibition, one strain changed from NA to WA, and three strains became non-adherent. For ¼ MIC90 applications, all treated strains remained non-adherent. For the effect of HP at ¼ MIC50, four of the seven strains that were initially WA became NA, and three strains remained classified as weakly adherent. In ¼ MIC90 applications, two of the three strains that were initially NA became WA.

### 3.4. WGS

The analysis of genomic mutations observed in variants of strains 32A–32A4, 181B–181B2, and 212A–212A4 is detailed in Table 3. Strain 32A4 presented a total of 13 mutations, of which 11 were simple and two were complex. The simple mutations affected genes such as *recF*, *gyrB*, *serS*, *rplI*, and *blaR1*, while complex mutations were found in the *mecR1* and *IS3 family transposase* genes. Strain 181B2 presented four mutations, three of which were simple, with changes in genes like *hsdR/M/S* and *ccrB*. Strain 212A4 had the highest number of complex mutations, with four genes (*dnaA*, *dnaN*, *recF*, and *gyrB*) affected by multiple mutations, resulting in five complex mutations and no simple mutations.

## 4. Discussion

It has been demonstrated that sanitizers, when applied under the conditions recommended by the manufacturers, are generally sufficient in reducing the risks caused by microbial growth [27]. However, improper sanitizing procedures, such as inadequate contact times on surfaces and failures in post-cleaning, can lead to the persistence of sanitizing solutions in sublethal doses in these environments [28]. Studies suggest that these stress conditions at sublethal concentrations may provoke responses that either favor or reduce the persistence of microorganisms, with varying responses observed for different pathogens [27].

When evaluating the efficiency of the sanitizers used in the MIC tests in this study, the HP values against *S. chromogenes* and *S. epidermidis* were around 1000 ppm, a concentration similar to that found by Santos et al. [29]; when evaluating the efficiency of HP against *S. aureus* strains over a contact time of 24 h, an MIC of 1400 ppm was obtained. As shown above, all strains of *S. chromogenes* and *S. epidermidis* that were exposed to sub-MICs of HP, showed growth after four exposures, while for OXA, only some of the strains showed growth. On the other hand, no strains grew after exposure to BAC at any of the concentrations. However, BAC proved to be the most efficient sanitizer for bacterial reduction, when compared to the reduction values for HP applications.

The findings obtained from different experimental contexts reinforce the antimicrobial efficacy of BAC against *S. aureus* and NAS, while also highlighting important limitations related to prolonged exposure and application methods. In topical formulations, such as hand sanitizers containing 0.1% BAC, significant growth inhibition was observed for *S. aureus*, *S. epidermidis*, *S. hominis*, and *S. haemolyticus*, confirming the potential of this compound as an effective antimicrobial agent for direct-contact sanitization [30]. However, when applied at sub-inhibitory concentrations, BAC induced increased MIC values and the selection of resistant mutants in both *S. aureus* and non-*aureus* species, including *S. pasteuri* and *S. xylosus*. Notably, this effect was counteracted when BAC exposure was combined with adjuvant strategies, such as toluidine blue-mediated photoinactivation, which effectively reduced bacterial loads by several orders of magnitude [31]. In clinical settings, chronic use of BAC-containing eye drops in glaucoma patients resulted in the isolation of *S. epidermidis* strains with elevated MICs and a higher frequency of the *qacC/smr* efflux pump gene, particularly among methicillin-resistant isolates, suggesting that selective pressure from long-term BAC exposure may contribute to the persistence of multidrug-resistant phenotypes [32]. Collectively, these findings indicate that, although BAC demonstrates substantial antimicrobial activity across a diverse range of staphylococcal species, inappropriate or continuous use may promote resistance development, emphasizing the need for proper dosing and the integration of complementary strategies to preserve its long-term effectiveness. A similar result is shown by Ríos-Castillo et al. [33], where tests of sanitizers formulated with HP at 4% and BAC at 1% showed satisfactory reductions (≥4 log10 of *S. aureus*) when bacterial reduction levels were evaluated immediately after application of the solutions, but when bacterial viability was evaluated at sanitization times after 24 h, BAC showed greater efficiency (≥3 log10 reduction), while HP showed reduction values of 0.94 log10, thus demonstrating greater stability in applications and antibacterial efficacy of BAC within 24 h.

The *S. chromogenes* 66B isolate was the most resistant to the sanitizer tests evaluated, with an MIC value for HP of 1500 ppm after sub-inhibition by OXA at ¼ MIC50 concentration (treatment 5). This relationship of decreased susceptibility between HP and OXA after serial sub-inhibition is demonstrated by Speck et al. [34], where after applying HP at final concentrations of 0.01% (*v*/*v*) against *S. aureus* (ATCC 29213 and ATCC 6538), a decrease in susceptibility to OXA is observed with at least a 16-fold increase in MIC values against the strains. The positive regulation of multidrug efflux pumps is among the mechanisms that can cause this reduction in sensitivity to OXA, a factor that can also be triggered in *S. aureus* strains if there are multiple exposures to other sanitizers such as BAC [35]. Similarly, multiple exposure to sub-MICs of β-lactam antibiotics can increase the virulence of MRSA [36]. In this study, strains of *S. epidermidis* 32A, 32B, 187B and 212A of *S. chromogenes* showed reduced sensitivity to BAC when exposed to OXA. Studies indicate that the attribution of borderline resistance to OXA may also be favored by the hyperproduction of compounds such as β-lactamases and the presence of the *blaZ* gene, responsible for the production of penicillin hydrolyzing substances [37,38]. Isolate 32B shows higher values for resistance to BAC and OXA, a fact that may be related to the presence of the *qacAB* gene, which is responsible for resistance to the quaternary ammonium compound (QAC) transported by plasmids and encoded by efflux pumps [4], in addition to presenting genes responsible for biofilm formation such as *ebps*, an adhesin, responsible for binding to host cells through its binding to elastin; the *cflA* gene, involved in adhesion and immune evasion; and the *eno* gene, which encodes α-enolase and is capable of binding to laminin, thus acting as a plasminogen receptor. In short, these biofilm genes are important in the initial phase of biofilm growth and adhesion, making it difficult for pathogens to die [39].

Mutations were observed in strain 32A in the *mecR1* and *IS3 family transposase* genes, both with multiple alterations, which may indicate a greater functional or adaptive impact. Mutations in *blaR1* and *mecR1*, two sensors of the resistance expression regulator system (*mecA*), are highly suggestive of the activation and reactivation of OXA resistance after the loss of resistance to this antibiotic [40]. Additionally, mutations were observed in the *gyrB* gene, which encodes subunit B of DNA gyrase, the enzyme targeted by quinolones. This suggests possible cross-resistance or non-classical resistance to this class of antibiotics [41]. The presence of multiple mutations in mobile genetic elements, such as the *IS3 transposase*, may indicate the mobilization of resistance genes and genomic plasticity. However, despite mutations in *blaR1* and *mecR1*, strain 32A is sensitive to OXA. This suggests that the mutations may be neutral, inactive, or compensated by other mechanisms [42]. Nevertheless, multiple resistance to CEF, TET, ERI, CLI, PEN, and EST suggests a multidrug-resistant profile, possibly favored by the presence of mobile elements (*IS3*) and accumulated mutations. The mutation in *gyrB* does not reflect resistance to quinolones (sensitivity to GEN and TOB), but it may have adaptive effects that are not evident in the tested phenotype.

The integration of genomic findings with the observed phenotypic profile provides insights into plausible molecular mechanisms underlying strain adaptation. Mutations in *blaR1* and *mecR1* suggest modulation of the signaling cascade responsible for *mecA* induction, which represents a central mechanism of β-lactam resistance in MRSA [43]. The identification of mutations in transposases and in the *ccrB* gene further supports the potential mobilization of mobile genetic elements, such as SCCmec, previously described as major drivers of resistance dissemination across clinical and environmental contexts [44]. Moreover, mutations in genes associated with DNA replication and repair (*dnaA*, *dnaN*, *recF*) and in *gyrB* indicate adaptive responses involving enhanced genomic plasticity, resembling what has been reported in *Staphylococcus* exposed to sub-inhibitory concentrations of fluoroquinolones, where both structural mutations in target genes and the overexpression of efflux pumps and regulators have been documented [45]. Recent studies have also demonstrated the association of disinfectant resistance genes (*qacA/B*, *smr*) with increased tolerance to sanitizers and multidrug-resistant profiles [46], supporting the hypothesis that exposure to sub-MIC levels of antibiotics and sanitizers can promote regulatory and genomic alterations that sustain the emergence of multidrug-resistant (MDR) phenotypes in *Staphylococcus* spp.

For strain 181B2, a notable mutation was found in the *ccrB* gene, which encodes a recombinase involved in the mobilization of the SCCmec chromosomal cassette. This genetic element often carries the *mecA* gene, which is responsible for OXA resistance. Although the *ccrB* mutation is not a direct marker of resistance, it suggests possible mobilization or rearrangement of SCCmec, that could affect the expression of resistance [47]. Mutations were also observed in the restriction/modification system (*hsdR*, *hsdM*, and *hsdS*), which can influence the entry of exogenous DNA and, indirectly, the acquisition of resistance genes. Despite the presence of *ccrB* mutations, the variant is sensitive to oxacillin, indicating that the SCCmec mobilization may not have included the *mecA* gene, or that the cassette is inactive. The only resistance detected was to ERI, which is not directly associated with the observed mutations. This suggests another mechanism is involved, such as an efflux pump [48].

Strain 212A4 has accumulated a large number of complex mutations in genes essential for DNA replication and repair, such as *dnaA*, *dnaN*, *recF*, and *gyrB*. Although it does not present direct mutations in classic beta-lactam resistance genes like *blaR1* or *mecR1*, the mutation pattern suggests an intense adaptive response to antimicrobial stress.

The mutation in *gyrB* may indicate potential resistance to quinolones, while multiple alterations in replication genes may reflect genomic instability or compensatory survival strategies [41]. Despite the absence of classic resistance genes, the 212A4 variant exhibits resistance to multiple antibiotics, especially β-lactams (CEF and PEN) and antimicrobials with different targets. The multiple mutations in replication and repair genes (especially *recF*, *dnaA*, and *gyrB*) indicate strong evolutionary adaptation. This may be due to the accumulation of compensatory or permissive mutations that confer this indirect multidrug resistance profile, even without the presence of *mecR1/blaR1* [49].

The increasing resistance of *S. aureus* and NAS to antimicrobials widely used in veterinary medicine, such as enrofloxacin and the combination of amoxicillin with clavulanic acid, represents a worrisome scenario that adds to the already high resistance rates to OXA reported in the literature. In avian isolates, resistance rates exceeded 80% for enrofloxacin and amoxicillin, with resistance to amoxicillin–clavulanate strongly associated with MDR profiles [50]. Similarly, NAS isolated from fermented foods also exhibited high resistance rates to this drug combination [51], reinforcing their role as potential reservoirs of transferable resistance genes. Furthermore, the use of enrofloxacin in animal production systems has been linked to the horizontal dissemination of resistance genes, thereby contributing to the expansion of MDR phenotypes in diverse environments [52]. Although studies on milk samples indicate lower resistance frequencies to amoxicillin–clavulanate [53], the overall trend across different contexts suggests a gradual reduction in the effectiveness of this antimicrobial. In companion animals, *S. aureus* isolates frequently showed resistance to enrofloxacin and other antimicrobial classes, highlighting the risk of transmission of resistant strains between animals and humans [54]. Thus, considering also the data obtained from food samples, a convergent resistance pattern emerges, underscoring the urgent need for integrated surveillance and prudent antimicrobial use in veterinary medicine, particularly within the framework of the One Health concept.

## 5. Conclusions

The results of this study demonstrate that exposure of *Staphylococcus* spp. strains to sub-inhibitory concentrations of sanitizers can induce phenotypic alterations in antimicrobial resistance associated with multiple genomic mutations. Notably, modifications were identified in classical regulatory genes of β-lactam resistance (*blaR1, mecR1*), in mobile elements related to SCCmec mobilization (*ccrB*, IS3 transposase), and in genes involved in DNA replication and repair (*dnaA, dnaN, recF, gyrB*), all of which suggest molecular adaptive mechanisms that go beyond a simple correlation between phenotype and mutation. These findings support the hypothesis that sub-MIC selective pressure favors not only the selection of resistant variants but also the activation of regulatory pathways and genomic plasticity that contribute to the emergence of multidrug-resistant phenotypes. Although further functional studies are required to confirm the specific role of these mutations, the present work provides initial evidence of possible molecular mechanisms linking sub-inhibitory sanitizer exposure to the evolution of antimicrobial resistance in *Staphylococcus* spp.

## Figures and Tables

**Table 1:** Concentrations of the antimicrobial agents applied in sub-MIC doses against the evaluated isolates of Non-*aureus Staphylococcus*.

Antimicrobial Agent	MIC 50 (ppm)	MIC 90 (ppm)	¼ MIC 50 (ppm)	¼ MIC 90 (ppm)
BAC	125	250	31.25 (Treatment 1)	62.5 (Treatment 2)
HP	1000	1000	250 (Treatment 3)	250 (Treatment 4)
OXA	0.027	0.108	0.00675 (Treatment 5)	0.027 (Treatment 6)

Legend = BAC: benzalkonium chloride; HP: sodium hypochlorite; OXA: oxacillin. MIC 50: concentration required for 50% inhibition of each strain evaluated; MIC 90: concentration required for 90% inhibition of each strain evaluated.

**Table 2:** Classification of biofilm formation in strains with and without oxacillin and sodium hypochlorite treatment.

Resistance Profile of Strains Before Sub-Inhibition		Sub-Inhibitory Treatment with HP				Sub-Inhibitory Treatment with OXA			
Isolated	Classification	¼ MIC50		¼ MIC90		¼ MIC50		¼ MIC90	
		Isolated	Classification	Isolated	Classification	Isolated	Classification	Isolated	Classification
19A	WA ^d^	19A1	NA ^a^	19A2	NA ^a^	-	-	-	-
32A	NA ^d^	32A1	NA ^a^	32A2	WA ^b^	32A3	WA ^d^	32A4 ^b^	NA
32B	WA ^e^	32B1	NA ^b^	32B2	NA ^a^	32B3	NA ^a^	32B4 ^b^	NA
41B	WA ^f^	41B1	NA ^c^	41B2	WA ^c^	-	-	-	-
66B	WA ^a^	66B1	NA ^c^	66B2	NA ^a^	66B3	NA ^b^	66B4 ^a^	NA
126B	NA ^f^	126B1	WA ^f^	126B2	NA ^a^	-	-	-	-
178B	NA ^f^	178B1	WA ^e^	178B2	WA ^d^	178B3	NA ^b^	-	-
181B	WA ^g^	181B1	WA ^d^	181B2	WA ^b^	-	-	-	-
212A	WA ^b^	212A1	WA ^d^	212A2	NA ^b^	212A3	NA ^c^	212A4 ^c^	NA
250B	WA ^c^	250B1	WA ^d^	250B2	NA ^b^	-	-	-	-

Legend = HP: sodium hypochlorite; OXA: oxacillin; NA: non-adherent; WA: weakly adherent. MIC 50: concentration required for 50% inhibition of each strain evaluated. MIC 90: concentration required for 90% inhibition of each strain evaluated. - = no growth of isolate after sub-inhibition. The biofilm formation rating is not reported for benzalkonium chloride (BAC), as there were no strains that survived sub-inhibition treatments using this sanitizer. Equal lowercase letters on the columns indicate significant equality by Tukey (*p* < 0.05).

**Table 3:** Results obtained for WGS for strains of *Staphylococcus* spp. after sub-inhibition tests with sanitizers and oxacillin.

Isolates	Mutation Position	Gene	Protein Name or Function	NCBI Library
32a–32a4	4276	*recF*	DNA replication/repair protein	ASM609437v1 ASM4594097v1 ASM1932966v1
		*gyrB*	DNA topoisomerase (ATP-hydrolyzing) subunit B	
	12,099	*serS*	Serine--tRNA ligase	ASM609437v1 ASM4594097v1 ASM1932966v1
		*sph*	Sphingomyelin phosphodiesterase	
	13,655	*sph*	Sphingomyelin phosphodiesterase	ASM609437v1 ASM4594241v1 ASM4594097v1
	20,005	*rplI*	50S ribosomal protein L9	ASM609437v1 ASM4594097v1 ASM1932966v1
	49,380	*blaR1*	Beta-lactam sensor/signal transducer BlaR1	ASM4594097v1 ASM4594241v1 ASM4594006v1
	58,217	*galU*	uncharacterized gene	ASM1932966v1
	93,331; 93,351	*mecR1*	Beta-lactam sensor/signal transducer MecR1	ASM1932966v1
	96,456	*cstB*	Uncharacterized gene	ASM1932966v1
	32,667	***-***	IS3 family transposase	ASM1932942v1
	42,855	***-***	DUF6038 family protein	ASM609437v1
	60,993	***-***	SdrD B-like domain-containing protein	ASM609437v1
	32,673; 32,823; 33,258; 33,273; 33,303	***-***	IS3 family transposase	ASM1932966v1
	42,855	***-***	DUF6038 family protein	ASM609437v1
	197,627	***-***	Metal ABC transporter solute-binding protein, Zn/Mn family	ASM609437v2
181B–181B2	33,653	*hsdR, hsdM, and hsdS*	Type I restriction endonuclease subunit R	ASM4594097v1
	76,949; 76,966; 77,007	***-***	DUF5906 domain-containing protein	ASM1932966v1
	77,402	*ccrB*	Cassette chromosome recombinase CcrB	ASM4594097v1 ASM4594006v1 ASM1932942v0
	77,648	*ccrB*	Cassette chromosome recombinase CcrB	ASM1932942v1 ASM1932966v ASM4594097v1
212a–212A4	86; 136; 138; 152; 159; 166; 186; 217; 229; 250; 256; 268; 276; 292; 343; 479; 694; 725; 734; 739; 776; 912	*dnaA*	Chromosomal replication initiator protein DnaA	ASM4857110v1 ASM1146687v1 ASM4857109v1
	1531; 1537 2072	*dnaN*	DNA polymerase III subunit beta	ASM1146687v1 ASM4857110v1 ASM4857108v1
	3372; 3378; 3387; 3438	***-***	DsbA family protein	ASM1146687v1
	4000; 4035; 4235; 4351	*recF*	DNA replication/repair protein RecF	ASM1146687v1 ASM4857110v1 ASM4857108v1
	5545	*gyrB*	DNA topoisomerase (ATP-hydrolyzing) subunit B	ASM1146687v1 ASM4857110v1 ASM4857108v1

## Data Availability

The original contributions presented in the study are included in the article and Supplementary Materials. Further inquiries can be directed to the corresponding author.

## Supplementary Materials

The following supporting information can be downloaded at: https://www.mdpi.com/article/10.3390/vetsci12100979/s1. Table S1: Results for sensitivity of *Staphylococcus* spp. strains prior to performing sub-inhibition testing with sanitizers and oxacillin. Table S2: Results for sensitivity of *Staphylococcus* spp. strains after sub-inhibition testing with sanitizers and oxacillin following CLSI comparative resistance parameters. Table S3: Results for sensitivity of *Staphylococcus* spp. strains after sub-inhibition testing with sanitizers and oxacillin following EUCAST comparative resistance parameters. Box S1: Determination of the Minimum Inhibitory Concentrations (MICs) for sodium hypochlorite (HP), benzalkonium chloride (BAC), and oxacillin (OXA) at different application times. MIC values are expressed in ppm.
