# Supplementary material for: Influence of Sub-Inhibitory Concentrations of Sanitizers and Oxacillin on the Resistance of Methicillin-Resistant Staphylococcus spp
Source: Vet Sci. 2025 Oct 11;12(10):979. doi: 10.3390/vetsci12100979 (PMC12567888; doi:10.3390/vetsci12100979)
Supplement: Supplementary file 1 [file vetsci-12-00979-s001.zip › vetsci-3861781-supplementary.pdf]

Influence of Sub-Inhibitory Concentrations of Sanitizers and Oxacillin on the Resistance of Methicillin-Resistant *Staphylococcus* spp.

Maria Eugênia Betim, Daniel Lucino Silva dos Santos, Thiago dos Santos Lopes, Bruna Lourenço Crippa, Érika Romão Bonsaglia, Stéfani Dantas, Vera Lúcia Mores Rall, Fernanda Buzzolla, Julia Arantes Galvão, Clarice Gebara, André Thaler, Nathália Cristina Cirone Silva

Table S1- Results for sensitivity of *Staphylococcus* spp. strains prior to performing sub-inhibition testing with sanitizers and oxacillin.

Strain	Species	GEN	OXA	CEF	TET	ERI	CLI	TOB	CLO	PEN	EST
19A	*S. epidermidis*	S	S	S	S	I	S	S	S	S	S
32A	*S. epidermidis*	S	S	S	R	R	R	S	R	R	S
32B	*S. epidermidis*	S	S	S	R	R	R	S	R	R	S
212A	*S. chromogenes*	S	S	S	S	S	S	S	S	S	S
41B	*S. chromogenes*	S	S	S	S	S	S	S	S	S	S
66B	*S. chromogenes*	S	S	S	S	S	S	S	S	S	S
126B	*S. epidermidis*	S	S	S	I	S	S	S	S	R	S
178B	*S. epidermidis*	S	S	S	S	S	S	S	S	S	S
181B	*S. epidermidis*	S	S	S	S	S	S	S	S	S	S
250B	*S. chromogenes*	S	S	S	S	S	S	S	S	S	S

*= R: antibiotic-resistant strain; S: antibiotic-sensitive strain; I: strains with intermediate action halos. GEN: gentamicin, OXA: oxacillin, CEF: cefoxitin, TET: tetracycline, ERI: erythromycin, TOB: tobramycin, PEN: penicillin, EST: streptomycin, CLI: clindamycin, and CLO: chloramphenicol.

Table S2- Results for sensitivity of *Staphylococcus* spp. strains after sub-inhibition testing with sanitizers and oxacillin following CLSI comparative resistance parameters.

STRAIN	GEN	OXA	CEF	TET	ERI	CLI	TOB	CLO	PEN	EST
19A1	S	S	S	R	S	S	S	S	R	S
32A1	S	S	R	S	R	R	S	S	S	R
32B1	S	S	R	R	R	R	S	S	R	R
41B1	S	S	R	R	R	S	S	S	S	S
66B1	S	S	R	S	S	S	S	S	S	S
126B1	S	S	R	R	S	R	S	S	S	S
178B1	S	S	R	S	R	R	S	S	R	R
181B1	S	S	R	S	R	R	S	S	R	R
212A1	S	S	R	R	S	S	S	S	R	S
250B1	S	S	S	S	S	S	S	S	S	S
19A2	S	S	S	S	R	S	S	S	S	S
32A2	S	S	R	R	R	R	S	S	R	R
32B2	S	S	R	R	R	R	S	S	R	R
41B2	S	S	S	S	S	S	S	S	S	S
66B2	S	S	S	S	S	S	S	S	S	S
126B2	S	S	S	S	R	S	S	S	S	S
178B2	S	S	S	S	R	S	S	S	S	S
181B2	S	S	S	S	R	S	S	S	S	S
212A2	S	S	R	R	R	S	S	S	R	S
250B2	S	S	S	S	S	S	S	S	S	S
32B3	S	S	R	R	R	R	R	R	R	R
32B4	S	S	R	R	R	R	R	R	R	R
212A3	S	S	R	R	R	R	S	S	R	S
212A4	S	S	R	R	R	R	S	S	R	S

*= R: antibiotic-resistant strain; S: antibiotic-sensitive strain; I: strains with intermediate action halos. GEN: gentamicin, OXA: oxacillin, CEF: cefoxitin, TET: tetracycline, ERI: erythromycin, TOB: tobramycin, PEN: penicillin, EST: streptomycin, CLI: clindamycin, and CLO: chloramphenicol. *S. epidermidis* (19A, 32A, 32B, 126B, 178B, 181B*)* and *S. chromogenes* (212A, 41B, 66B, 250B*).* Strains were classified according to the treatment they received. Strains with codes ending in 1 were treated with the MIC50 of sodium hypochlorite (HP), while those ending in 2 were treated with the MIC90. Similarly, strains with codes ending in 3 were treated with the MIC50 of oxacillin OXA, and those ending in 4 were treated with the MIC90.

Table S3- Results for sensitivity of *Staphylococcus* spp. strains after sub-inhibition testing with sanitizers and oxacillin following EUCAST comparative resistance parameters.

STRAIN	GEN	OXA	CEF	TET	ERI	CLI	TOB	CLO	PEN	EST
19A1	S	S	S	S	R	S	S	S	R	R
32A1	R	R	S	R	R	R	S	R	R	R
32B1	R	R	R	R	R	R	S	R	R	R
41B1	S	R	S	S	S	S	S	S	S	R
66B1	S	R	S	S	S	S	S	S	S	R
126B1	S	S	S	S	R	S	R	S	S	R
178B1	S	S	S	S	S	S	R	R	S	R
181B1	S	S	S	S	R	S	S	S	R	R
212A1	S	R	R	S	R	S	S	S	R	R
250B1	S	S	S	S	S	S	S	S	S	R
19A2	S	S	S	S	R	S	S	S	R	R
32A2	R	R	S	R	R	R	S	R	R	R
32B2	R	R	R	R	R	R	S	R	R	R
41B2	S	R	S	S	S	S	S	S	S	R
66B2	S	S	S	S	S	S	S	S	S	S
126B2	S	S	S	S	R	S	R	S	R	R
178B2	S	S	S	S	R	S	R	R	S	R
181B2	S	S	S	S	R	S	S	S	R	R
212A2	S	R	R	S	R	S	S	S	R	R
250B2	S	R	S	S	S	S	S	S	R	R
32B3	R	R	R	R	R	R	R	R	R	R
32B4	R	R	R	R	R	R	R	R	R	R
212A3	S	R	R	S	R	S	S	S	R	R
212A4	S	R	R	S	R	S	S	S	R	R

*= R: antibiotic-resistant strain; S: antibiotic-sensitive strain; I: strains with intermediate action halos. GEN: gentamicin, OXA: oxacillin, CEF: cefoxitin, TET: tetracycline, ERI: erythromycin, TOB: tobramycin, PEN: penicillin, EST: streptomycin, CLI: clindamycin, and CLO: chloramphenicol. Strains were classified according to the treatment they received. *S. epidermidis* (19A, 32A, 32B, 126B, 178B, 181B) and *S. chromogenes* (212A, 41B, 66B, 250B). Strains with codes ending in 1 were treated with the MIC50 of sodium hypochlorite (HP), while those ending in 2 were treated with the MIC90. Similarly, strains with codes ending in 3 were treated with the MIC50 of OXA, and those ending in 4 were treated with the MIC90.

Box S1- Determination of the Minimum Inhibitory Concentration (MIC) for sodium hypochlorite (HP), benzalkonium chloride (BAC), and oxacillin (OXA) at different application times. MIC values are expressed in ppm.

**Antimicrobial**		**HP**			**BAC**			**OXA**
**Time (min)**		**5**	**10**	**15**	**5**	**10**	**15**	**-**
**Isolate**	19A	125^{c}	93.8^{c}	46.9^{b}	125^{d}	125^{f}	145.8^{h}	0.000105^{a}
	32A	83.3^{a}	62.5^{a}	41.7^{a}	83.3^{c}	62.5^{c}	62.5^{e}	0.003586^{e}
	32B	125^{c}	125^{e}	125^{f}	125^{d}	62.5^{c}	62.5^{e}	0.013553^{f}
	126B	156.3^{e}	93.8^{c}	187.5^{g}	187.5^{f}	62.5^{c}	93.8^{g}	0.000105^{a}
	178B	125^{c}	104.2^{d}	104.2^{e}	83.3^{c}	93.8^{d}	41.7^{d}	0.000105^{a}
	181B	166.7^{f}	125.0^{e}	83.3^{c}	125.0^{d}	104.2^{e}	72.9^{f}	0.000316^{c}
	41B	145.8^{d}	83.3^{b}	83.3^{c}	62.5^{a}	52.1^{b}	26.0^{b}	0.000105^{a}
	66B	145.8^{d}	93.8^{c}	41.7^{a}	72.9^{b}	31.3^{a}	23.4^{a}	0.000105^{a}
	212A	104.2^{b}	104.2^{d}	93.8^{d}	62.5^{a}	52.1^{b}	41.7^{d}	0.001740^{d}
	250B	208.3^{g}	104.2^{d}	104.2^{e}	166.7^{e}	62.5^{c}	36.5^{c}	0.000158^{b}

=Strains were classified according to the treatment they received. *S. epidermidis* (19A, 32A, 32B, 126B, 178B, 181B*)* and *S. chromogenes* (212A, 41B, 66B, 250B). Equal lowercase letters on the columns indicate significant equality by Tukey ($P < 0.05$).
